# Timing of Wnt Inhibition Modulates Directed Differentiation of Medial Ganglionic Eminence Progenitors from Human Pluripotent Stem Cells

**DOI:** 10.1155/2018/3983090

**Published:** 2018-06-27

**Authors:** Ivanna Ihnatovych, Alexandra Lew, Evelyn Lazar, Anna Sheng, Timot Kellermayer, Kinga Szigeti

**Affiliations:** Department of Neurology, State University of New York at Buffalo, Buffalo, NY, USA

## Abstract

*In vitro* differentiation of human pluripotent stem cell into relevant cell types is a desirable model system that has the human biological context, is a renewable source, and is scalable. GABA interneurons and basal forebrain cholinergic neurons, derivates of the medial ganglionic eminence (MGE), are implicated in diverse neuropsychiatric diseases. Various protocols have been proposed to generate MGE progenitors: the embryoid body- (EB-) based rosette-derived (RD), the adherent (AdD), and the nonadherent (NAdD) approaches. While Wnt inhibition is frequently incorporated into the strategy, the timing varies between protocols and there is a lack of standardized outcome reporting, which precludes direct comparison. Here, we report a head-to-head comparison in three distinct experimental models to establish whether Wnt inhibition during neural stem cell, NSC (stage 1), or neural progenitor cell, NPC (stage 2), formation facilitates MGE differentiation. Wnt inhibition at both stages promotes MGE progenitor differentiation when compared to no inhibition. However, NSC (stage 1) Wnt inhibition markedly reduces the number of MGE progenitors available for downstream applications in the RD and the NAdD protocols due to early inhibition of proliferation. NPC (stage 2) Wnt inhibition in the adherent system is comparable to the EB-based methods offering a techically less challenging alternative.

## 1. Introduction

Medial ganglionic eminence (MGE) progenitors arise from the ventral forebrain (reviewed in [1, 2]) and serve as precursors to GABA interneurons and basal forebrain cholinergic neurons (BFCNs). Dysfunction of GABA interneurons has been implicated in epilepsy, schizophrenia, autism, and intellectual disabilities (reviewed in [3]), while BFCN malfunction/degeneration is associated with Alzheimer's disease (AD) [4], Huntington's disease [5], dementia with Lewy bodies [6], and Parkinson's disease [7]. Therefore, if feasible and reproducible *in vitro* differentiation protocols are developed, these relevant neuronal phenotypes could serve as model systems to study disease mechanisms, identify functional readouts for high-throughput screens, and develop treatment strategies for neuropsychiatric diseases. Using iPSC derived from mutation carriers, this model system can complement genetic association studies for functional characterization of mutations/variants and elucidate the cell type-specific mechanism in Mendelian diseases.

*In vitro* differentiation of hESC/iPSC into specific types of neurons involves neural induction and patterning first along the rostro-caudal (R-C) and subsequently along the dorso-ventral (D-V) neuraxes, mimicking the embryological developmental processes. R-C patterning of the CNS consists of expansion of the neural tube and demarcation of the forebrain, midbrain, hindbrain, and spinal cord, followed by patterning of the telencephalon along the D-V neuraxis. R-C and D-V patterning is driven by morphogen expression in a regulated temporal and spatial distribution. The most studied morphogens are bone morphogenic proteins (BMPs), fibroblast growth factors (FGFs), and Wnt proteins. Similar to *in vivo* differentiation, regional identity of neural progenitors *in vitro* is determined by the gradient of morphogens with opposing effects (reviewed in [8]).

Wnt signaling is crucial for regulation of heterogeneity and regional identity of neural progenitor cells (NPC) *in vitro* [9, 10]. Low concentration of Wnt is needed for formation of the rostral brain, intermediate concentration of Wnt leads to the midbrain differentiation, and high concentration of Wnt is important to hindbrain and spinal cord differentiation [10]. Early inhibition of the Wnt/*β*-catenin pathway is required for telencephalic induction of the neural plate (reviewed in [11]). Treatment of hESC-derived neuroectodermal cells with DKK1 or XAV939 (Wnt inhibitors) enhances telencephalic commitment [12–14], and IWP-2- (Wnt inhibitor-) treated hESCs express more forebrain markers (PAX6 and OTX2) than untreated hESCs do [9]. Induction of ventral identities is determined by coordination of Sonic hedgehog (SHH) and Wnt signaling [15]. High concentration of SHH and low concentration of Wnt lead to formation of the ventral forebrain (reviewed in [1, 2]).

As MGE progenitors arise from the most ventral part of the forebrain, inhibition of Wnt signaling is frequently incorporated into the differentiation protocols. In addition, various technical approaches have been used: (1) the EB-based differentiation protocol with rosette formation/selection [15–17] or without it [13, 18, 19], (2) the adherent protocol, AdD [12, 20], and the EB-based nonadherent differentiation protocol, NAdD [21]. Most of the studies that utilized either the EB-based without rosette formation or the AdD differentiation protocols used Wnt inhibitors (DKK1, IWP-2, and XAV939) in combination with dual SMAD inhibition [22] during the neural stem cell (NSC) stage to induce neural differentiation of hESCs and enhance rostralization of the telencephalon [12, 13, 18–20]. Ventralization of the neuroepithelia was achieved by treatment of the cells with SHH in combination with small molecule purmorphamine, PUR [12, 13, 20], or with FGF8 [18, 19]. In contrast, inhibition of Wnt signaling during the stage of neuroepithelia ventralization (NPC stage) resulted in a significant increase of MGE progenitors in the EB-based rosette-derived protocol [15]. The NAdD protocol is distinctly different as only SMAD inhibition without Wnt inhibitors or exogenous SHH is required to achieve ventral forebrain identity [21].

**Figure 1:**
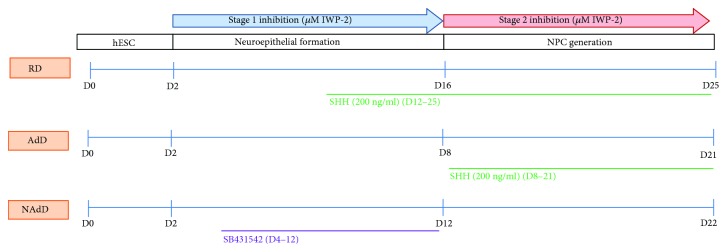
Schematic outline of timing of Wnt inhibition in MGE progenitor formation: head-to-head comparison of the three protocols.

Experimental strategies vary in methodology at multiple levels including conditions of the cells undergoing differentiation (adherent versus nonadherent cells); composition of differentiation media and supplements, exogenous SHH, its concentration and timing, or whether it was applied at all; and timing of Wnt inhibition or whether it was used. We present a head-to-head comparison of stage 1 (NSC) and stage 2 (NPC) Wnt inhibition in three distinct experimental models (Figure 1) keeping other variables as originally reported [17, 21, 23] to establish whether inhibition during NSC or NPC formation facilitates MGE differentiation within each system. Finally, as purity determines the signal-to-noise ratio in downstream applications, purity of MGE progenitor culture is reported for each protocol.

## 2. Methods

### 2.1. hESC (H9) Culture

H9 cells were cultured in 6-well plates coated with Matrigel (1 : 70 Matrigel (Corning): DMEM/F-12 Glutamax (Life Technologies)) in Essential 8 medium with E8 supplement (all Thermo Fisher). Cells were maintained at 37°C/5% CO_2_. Cultures were fed every day. Cells were subcultured every 4–6 days using Dispase (Life Technologies) or ReLeSR (Stem Cell Technologies) until colonies reached maximum size. Spontaneously differentiating cells were manually removed.

### 2.2. Differentiation Protocols

#### 2.2.1. RD Protocol

This protocol was based on Liu et al. [17] with modifications. Undifferentiated H9 (hES) cells were detached at day 0 and continued to float in T-25 flasks with Essential 6 medium (E6 medium) (Life Technologies) on a slow rotary shaker. H9 cells formed embryoid bodies (EB) at day 2. The medium was replaced with neural induction medium (NIM) [DMEM/F-12 + Glutamax, N2 (Life Technologies), nonessential amino acids (Gibco), heparin (Stem Cell Technonlogies), and pen/strep] on day 4. EB were attached in 6-well plates on day 7, and by day 10 neural rosettes were present, indicating the development of primitive neuroepithelia. Ventralization of primitive neuroepithelia was started at day 10 by adding SHH (200 ng/ml). On day 16, neural tube-like rosettes were detached and transferred into T-25 flasks in NIM with 2% B27 (Life Technologies) to form neurospheres. The cultures were fed every other day. On day 25, the neurospheres were characterized. Remaining neurospheres were collected, dissociated with Accutase (Stem Cell Technologies), and plated onto 6-well plates for further expansion.

#### 2.2.2. AdD Protocol

Adherent differentiation protocol has been described previously [23]. Neural induction began at day 0 by adding Gibco pluripotent stem cells (PSC) to neural induction medium (NIM) [Neurobasal Medium (Life Technologies) containing Neural Induction Supplement (Gibco)] to 20–30% confluent undifferentiated hES cells. The medium was changed every other day, and nonneural cells were removed from the cultures. On day 7, the primitive neuroepithelia were formed, dissociated with Accutase (Life Technologies), and plated on coated dishes for further expansion. Cultures were fed every other day with NSC expansion medium (NEM) [Neurobasal Medium (Life Technologies), DMEM/F-12 (Life Technologies), and Neural Induction Supplement (Gibco)]. Expanded NSC at passage 2 (P2) were further ventralized by adding SHH (R&D Systems) every other day from day 7 to day 21. At day 21, NPCs were characterized.

#### 2.2.3. NAdD Protocol

Nonadherent protocol was adapted from Crompton et al. [21]. Undifferentiated hESC colonies were detached at day 0 and transferred in E6 medium to T-25 flasks on the rotary shaker. The colonies remained floating throughout the duration of the experiment. By day 2, EBs were observed in the culture. On day 4, the medium was replaced with NIM [DMEM/F-12 + Glutamax, 1% N2 (Life Technologies), nonessential amino acids (Gibco), heparin (Stem Cell Technologies), and pen/strep]. On day 12, neuroepithelia structures appeared (confirmed by ICC and qPCR) and the medium was changed to NEM [DMEM/F-12 with Glutamax, 1% penicillin, and streptomycin, supplemented with 1% N2 and 2% B27 (all Life Technologies), plus FGF2 and EGF (20 ng/ml) and heparin (5 *μ*g/ml)]. NSC were fed every other day. At day 22, NPCs were characterized.

#### 2.2.4. Treatment with Inhibitors

Wnt inhibitor IWP-2 (2 *μ*g/ml) was added during either neuroepithelia/NSC formation (stage 1) or NPC formation (stage 2) as marked in the experimental protocols (Figure 1). Nodal/TGF-*β* signaling inhibitor SB431542 (20 *μ*M) was added from day 4 until day 12 in the NAdD protocol.

### 2.3. Quantitative Polymerase Chain Reaction (qPCR)

Total RNA was isolated from cell cultures using TRIzol (Invitrogen) according to the manufacturer's protocol. cDNA was synthesized from 500 ng of total RNA using ImProm-II Reverse Transcriptase (Promega) and oligo (DT) (Promega). The reaction was carried out at 42°C for one hour. For quantitative gene expression, standard RT-qPCR was performed using the primers (*IDT*) listed in Supplementary data, Table S1. qPCR was performed using the SYBR Green Master Mix (Biotool) and run on a Bio-Rad CFX Connect cycler (Bio-Rad). Samples were assayed with 3 technical replicates, and data was analyzed using the ΔΔC_T_ method and normalized to GAPDH expression. Data are presented as the average of the replicates ± standard error of the mean.

### 2.4. Immunocytochemistry (ICC) and Confocal Microscopy

Cells plated on 8-well glass chambers (Thermo Fisher) were fixed with 4% paraformaldehyde (Mallinckrodt Baker) for 15 minutes, permeabilized with 0.1% Triton X100 (Mallinckrodt Baker) for 10 minutes, and blocked with blocking buffer (5% BSA in PBS) for 1 hour at room temperature (RT). Cells were incubated overnight at 4°C with primary antibodies (Supplementary data, Table S2). On the next day, cells were incubated for 1 h at RT with secondary antibodies. Both primary and secondary antibodies were diluted in blocking buffer. Slides were mounted with a ProLong® Gold Antifade reagent with DAPI (Life Technologies), and confocal images were captured by using a LSM 510 Meta microscope (40x objective). Images were acquired using ZEN Black software. Counting of NKX2.1^+^ and PAX 6^+^ cells was performed by two independent raters in a blinded fashion. For each condition, at least three images with at least 100 cells per image were counted. The NKX2.1/PAX6 ratio was calculated based on cell counts for each condition. The experiments were conducted in triplicates. Purity of the hESC-derived MGE progenitor population was assessed by NKX2.1^+^ and PAX6^+^ cell count in ICC images. It was determined by the ratio of NKX2.1^+^ cells divided by the total number of generated NPC (NKX2.1^+^ and PAX6^+^ cells).

### 2.5. SHH ELISA

Cultured media were collected at D2, D4, and D12 of neural differentiation under the NAdD protocol and stored at −80°C. Concentration of SHH in the media was estimated using a human-specific ELISA kit (Abcam, cat. number ab100639) according to the manufacturer's protocol.

### 2.6. Statistical Analysis

Values are expressed as means ± SD or ± SEM, as indicated in figure legends.

Statistical significance was determined by an unpaired Student *t*-test (two-tailed). *p* values less than 0.05 were deemed statistically significant.

## 3. Results

**Figure 2:**
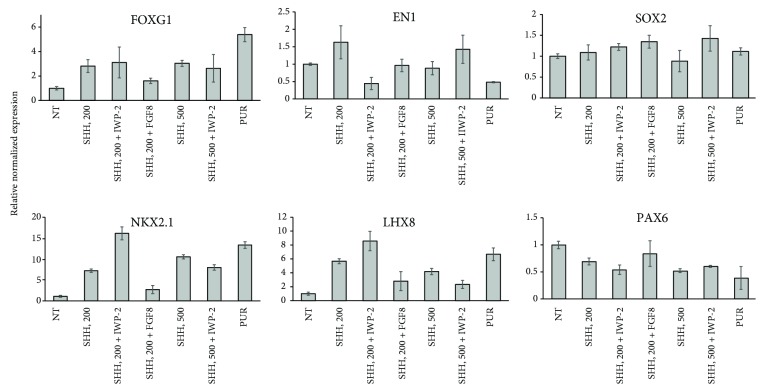
Gene expression analysis of NPC (D21) generated by the AdD protocol. NSC (D7) were treated for 14 days with either SHH alone, 200 ng/ml SHH (SHH, 200), 500 ng/ml SHH (SHH, 500), or in combination with 100 ng/ml FGF8 (SHH, 200 + FGF8) or 2 mM IWP-2 (SHH, 200 + IWP-2; SHH, 500 + IWP-2), and with 1.5 mM purmorphamine (PUR).

Preliminary experiments were conducted to address the following questions: which concentration of SHH is optimal for generation of MGE progenitors? Is FGF8 needed for MGE generation? Does Wnt inhibition facilitate the MGE fate? Can SHH be substituted with PUR? The AdD differentiation protocol was employed: neuroepithelia (D7) were treated for 14 days with either SHH alone at a concentration of 200 ng/ml (SHH, 200) or 500 ng/ml (SHH, 500) or in combination with FGF8, 100 ng/ml (SHH, 200 + FGF8), or with a Wnt inhibitor, IWP-2 (2 *μ*M). At D21, NPCs were collected for gene expression analysis (Figure 2). The conclusions of the preliminary studies are as follows: (1) Cotreatment of SHH with FGF8 is not necessary, (2) SHH can be substituted with PUR, and (3) expression of ventral markers (*NKX2.1* and *LHX8*) is the highest and expression of a dorsal marker (*PAX6*) is the lowest in the groups either treated with high concentration of SHH (SHH, 500) or PUR or cotreated with IWP-2 (SHH, 200 + IWP-2). The effect of IWP-2 is the highest in the context of 200 ng/ml SHH as compared to 500 ng/ml SHH, and it is higher than 500 ng/ml SHH alone. As the current study is aimed at demonstrating the importance of timing of Wnt inhibition for generation of MGE progenitors, the concentration of SHH 200 ng/ml was chosen for subsequent experiments.

A head-to-head comparison of stage 1 and stage 2 Wnt inhibition was performed in three distinct experimental systems. A schematic timeline of the RD, AdD, and NAdD protocols depicts stages of MGE progenitor generation and time of treatment with IWP-2, SHH, or SB431542 (Figure 1).

### 3.1. Rosette-Derived Protocol

**Figure 3:**
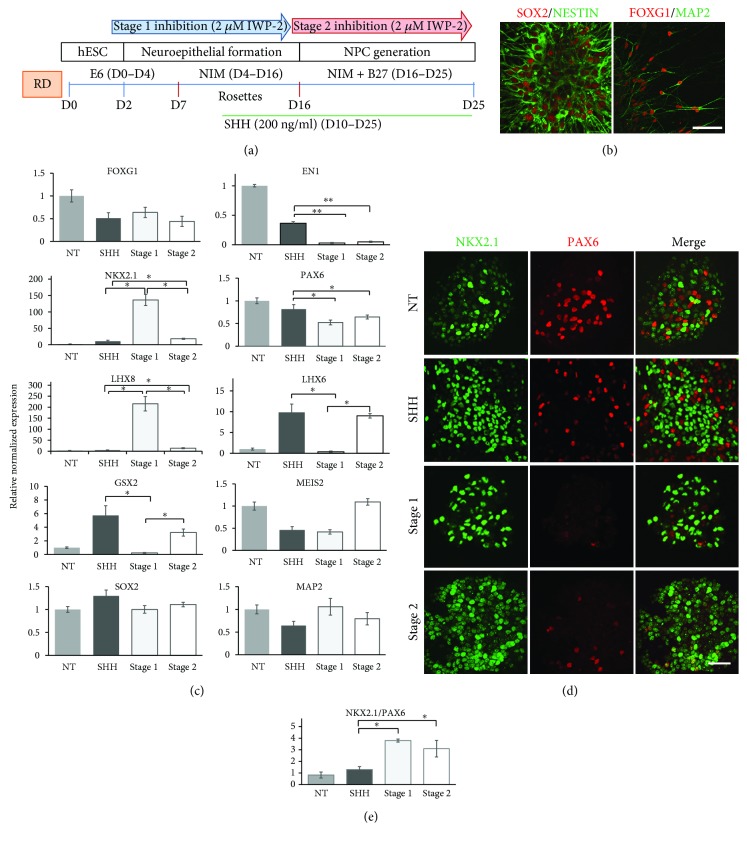
Effect of timing of Wnt inhibition on generation of MGE progenitors in the RD protocol. (a) Schematic timeline of the RD protocol showing stages of MGE progenitor generation, involved techniques, medium composition, and time of treatment with SHH and IWP-2 and medium change. (b) Confocal images of NPC double stained with SOX2/NESTIN and FOXG1/MAP2 antibodies. NPC collected at D25 and plated on 8-well chambers were fixed and stained two days later. Scale bar: 100 *μ*M. (c) Quantitative RT-PCR analysis of various markers (expression relative to nontreated cells, NT = 1) in NPC on D25 of directed differentiation. Data are presented as mean ± SEM. ^∗^ *p* < 0.05 and ^∗∗^ *p* < 0.001 difference between treatment with SHH alone and SHH supplemented with Wnt inhibition during NSC stage (stage 1) and/or NPC stage (stage 2) and between Wnt inhibition in stage 1 and stage 2. (d) Immunocytochemistry analysis of NPC for ventral, NKX2.1, and dorsal, PAX6, forebrain markers in response to treatment with SHH alone and SHH supplemented with Wnt inhibition during NSC stage (stage 1) and/or NPC stage (stage 2). Scale bar: 100 *μ*M. (e) Quantification of data in (d). Graph showing the NKX2.1/PAX6 ratio in untreated cells (NT) and cells treated with SHH alone and SHH supplemented with Wnt inhibition during the NSC stage (stage 1) and/or NPC stage (stage 2). Data are presented as mean ± SD. ^∗^ *p* < 0.05 difference between treatment with SHH alone and with SHH + IWP-2.

First, timing of Wnt inhibition was analyzed in the RD protocol, a gold standard differentiation protocol for NPC generation in general [24] and for MGE progenitors in particular [17].  Figure 3(a) schematically depicts the timeline of NPC generation, medium change, and time of treatment with SHH and a Wnt inhibitor, IWP-2. The protocol involves the following steps: detachment of hESC (high-quality colonies) from matrigel (D0), formation of embryoid bodies (EB) (by D2), attachment of EB followed by rosette formation—neuroepithelium development (D7–D16)—and, finally, selection of rosettes and generation of neurospheres/neural progenitor cells (NPC) (Figure 3(a)). This strategy results in a pure population of NPC which homogenously express pan neuronal markers SOX2 and NESTIN (Figure 3(b)) and demonstrate forebrain identity as verified by FOXG1/MAP-2 staining (Figure 3(b)).

Regional identity of NPC along the R-C axis was characterized by gene expression of forebrain (*FOXG1*), midbrain (*EN1*), and hindbrain (*HOXC6*) markers. The results revealed that (1) treatment with SHH alone or with SHH and IWP-2 neither during neuroepithelium formation (stage 1) nor during NPC generation (stage 2) affected the expression of *FOXG1* (Figure 3(c), top row; Supplementary data, Table S3). *FOXG1* expression was high in nontreated (NT) cells. (2) In contrast, *EN1* expression declined in the cells treated with SHH alone compared to NT cells and treatment with IWP-2 (at both stage 1 and stage 2) decreased it further (Figure 3(c), top row; Supplementary data, Table S3). (3) Expression of hindbrain marker *HOXC6* was low in nontreated cells and further downregulated after treatment with SHH and/or IWP-2 (Supplementary data, Figure S1).

Neuronal identity along the D-V axis was characterized by expression of dorsal (*PAX6*) and ventral (*NKX2.1*) forebrain markers. RT-qPCR data showed that the expression level of *PAX6* was the highest in untreated cells; treatment with SHH alone and in combination with IWP-2 during both stages of Wnt inhibition significantly decreased *PAX6* expression (Figure 3(c), second row; Supplementary data, Table S3). In contrast, expression of *NKX2.1* increased 5 times in the cells treated with SHH alone when compared to nontreated controls (Figure 3(c), second row; Supplementary data, Table S3). Treatment of cells with IWP-2 during neuroepithelium formation (stage 1) led to a further 12.4-fold increase in *NKX2.1* expression in comparison to SHH alone, while inhibition of the Wnt pathway during NPC generation (stage 2) resulted in only a 1.7-fold elevation of the ventral marker expression (Figure 3(c), second row; Table S3). In addition to *NKX2.1*, expression of two other MGE markers, *LHX6* and *LHX8*, was assessed. The expression profile of *LHX8* was similar to that of *NKX2.1* with an almost 45-fold increase with Wnt inhibition in stage 1 and an only 2.8-fold increase with Wnt inhibition in stage 2 when compared to the treatment with SHH alone (Figure 3(c), third row; Supplementary data, Table S3). In contrast, expression of *LHX6* was 30 times lower in the cells treated with IWP-2 during neuroepithelium formation/dorsalization (stage 1) and was equal to the SHH only-treated cells if Wnt inhibition occurred in stage 2 (Figure 3(c), third row; Supplementary data, Table S3). The opposite effect of stage 1 inhibition on *LHX6* and *LHX8* expression levels might be of great significance as transcription factor LHX8 is crucial for further differentiation of MGE-derived BFCN [25], whereas LHX6 is important for GABA interneuron neurogenesis [26].

Markers of the lateral ganglionic eminence (LGE), *GSX2* and *MEIS2*, were measured. While Wnt inhibition at both stage 1 and stage 2 led to a significant decrease in *CSX2* expression compared to treatment with SHH alone, the effect of stage 1 inhibition was more prominent. On the other hand, treatment of the cells with IWP-2 at both stages of neural development did not have any effect on *MEIS2* expression (Figure 3(c), fourth row; Supplementary data, Table S3). As expected, Wnt inhibition did not alter the expression of a pan neuronal marker, *SOX2*, or the marker of postmitotic neuron, *MAP-2* (Figure 3(c), bottom row; Supplementary data, Table S3).

MGE progenitors are defined as NKX2.1^+^/PAX6^−^ forebrain progenitors. NKX2.1 and PAX6 expression at the protein level was assayed by ICC and quantified by PAX6^+^ and NKX2.1^+^ cell count (Supplementary data, Figure S2). Representative confocal images of the nontreated cells (NT), cells treated with SHH alone (D10–D25), and those treated with SHH and IWP-2 in stage 1 (D2–D12) or with SHH and IWP-2 in stage 2 (D12–D22) are shown (Figure 3(d)). Immunostaining demonstrated a decrease in the number of PAX6^+^ cells after treatment with SHH alone and a further marked decrease in PAX6^+^ cells after IWP-2 in stage 1 and stage 2, compared to untreated controls (Figure 3(d)). The NKX2.1/PAX6 ratio equaled 0.83 in untreated cells (NT), but increased to 1.3 in the cells treated with SHH alone. Inhibition of Wnt signaling during either neural induction/rostralization (stage 1) or ventralization of neuroepithelia (stage 2) increased this ratio to 3.8 and 3.1, respectively, suggesting that over 75% of the NPC culture are MGE progenitors (Figure 3(e)).

### 3.2. Adherent Protocol

**Figure 4:**
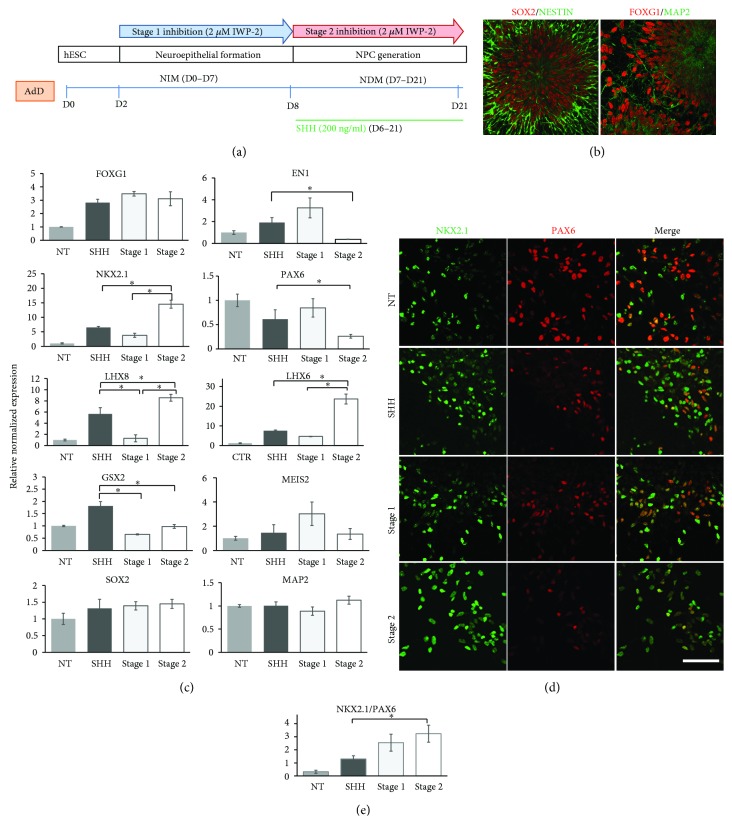
Effect of timing of Wnt inhibition on generation of MGE progenitors in the AdD protocol. (a) Schematic timeline of the AdD protocol showing stages of MGE progenitor generation, involved techniques, medium composition, and time of treatment with SHH and IWP-2 and medium change. (b) Confocal images of NPC double stained with SOX2/NESTIN and FOXG1/MAP2 antibodies. NPC collected at D25 and plated on 8-well chambers were fixed and stained two days later. Scale bar: 100 *μ*M. (c) Quantitative RT-PCR analysis of various markers (expression relative to nontreated cells, NT = 1) in NPC on D25 of directed differentiation. Data are presented as mean ± SEM. ^∗^ *p* < 0.05 difference between treatment with SHH alone and SHH supplemented with Wnt inhibition during the NSC stage (stage 1) and/or NPC stage (stage 2) and between Wnt inhibition in stage 1 and stage 2. (d) Immunocytochemistry analysis of NPC for ventral, NKX2.1, and dorsal, PAX6, forebrain markers in response to treatment with SHH alone and SHH supplemented with Wnt inhibition during NSC stage (stage 1) and/or NPC stage (stage 2). Scale bar: 100 *μ*M. (e) Quantification of data in (d). Graph showing the NKX2.1/PAX6 ratio in untreated cells (NT) and cells treated with SHH alone and SHH supplemented with Wnt inhibition during NSC stage (stage 1) and/or NPC stage (stage 2). Data are presented as mean ± SD. ^∗^ *p* < 0.05 difference between treatment with SHH alone and with SHH + IWP-2.

We examined the purity of MGE progenitors obtained through the adherent protocol (AdD).  Figure 4(a) schematically shows the time course of MGE progenitor differentiation, medium change, treatment with SHH, and timing of Wnt inhibition. The generation of NPC takes 21 days and requires only the change of media with specific factors/inhibitors, but does not involve technically challenging manipulations. Similarly to the RD approach, the AdD protocol results in a pure population of SOX/NESTIN- and FOXG1/MAP2-positive neuronal progenitors (Figure 4(b)).

In the AdD strategy, forebrain (*FOXG1*), midbrain (*EN1*), and hindbrain (*HOXC6*) markers are expressed in untreated cells, NT (Figure 4(c), top row, Supplementary data, Figure S1 and Table S3). In cultures treated with SHH alone and with SHH and IWP-2 during stage 1 and IWP-2 during stage 2, expression of the forebrain marker *FOXG1* increased. Meanwhile, expression of *EN1* significantly decreased in the cells treated with IWP-2, stage 2 (Figure 4(c), top row), when compared to the SHH-treated cells. Wnt inhibition during both stages of NPC generation led to undetectable *HOXC6* expression (Supplementary data, Figure S1).

Inhibition of Wnt signaling resulted in considerable differences in expression of ventral markers *NKX2.1*, *LHX8*, and *LHX6*. Treatment of the cells with IWP-2 while ventralizing neuroepithelia (stage 2) caused a 2.5-fold increase in *NKX2.1* expression (Figure 4(c), second row; Supplementary data, Table S3) and a 1.5- and a 3.5-fold increase in *LHX8* and *LHX6* expressions, respectively, compared to a level achieved by treatment with SHH alone (Figure 4(c), third row; Supplementary data, Table S3). Interestingly, treatment of the cells with IWP-2 during neural induction/rostralization (stage 1) did not change the expression of *NKX2.1* and *LHX6*, but decreased the expression of *LHX8*. Treatment of neuroepithelia with SHH alone downregulated the expression of dorsal marker *PAX6* compared to untreated controls; stage 2 Wnt inhibition further enhanced this effect (Figure 4(c), second row; Supplementary data, Table S3). Wnt inhibition during either stage 1 or stage 2 did not change the expression of LGE marker *MEIS2*, but slightly decreased the expression of *GSX2* (Figure 4(c), fourth row; Supplementary data, Table S3). As expected, the expression levels of *SOX2* and *MAP2* were not affected by IWP-2 treatment either (Figure 4(c), bottom row; Supplementary data, Table S3).

To verify if changes in gene expression translate into protein expression, immunostaining followed by confocal microscopy and counting of NKX2.1^+^ and PAX6^+^ cells was performed. ICC analysis revealed that while majority of untreated cells are dorsal forebrain progenitors (PAX6^+^ cells), treatment with SHH alone and in combination with IWP-2 treatment, especially during stage 2, leads to significant ventralization of neuroepithelia as shown by increased NKX2.1^+^ cell count (Figure 4(d), Supplementary data, Figure S2). The NKX2.1/PAX6 ratio increased from 0.3 (in untreated controls, NT) to 1.2 in cells with SHH alone and to 2.55 in cells treated with IWP-2 in stage 1 and to 3.2 in cells treated with IWP-2 in stage 2 (Figure 4(e)).

### 3.3. Nonadherent Protocol

**Figure 5:**
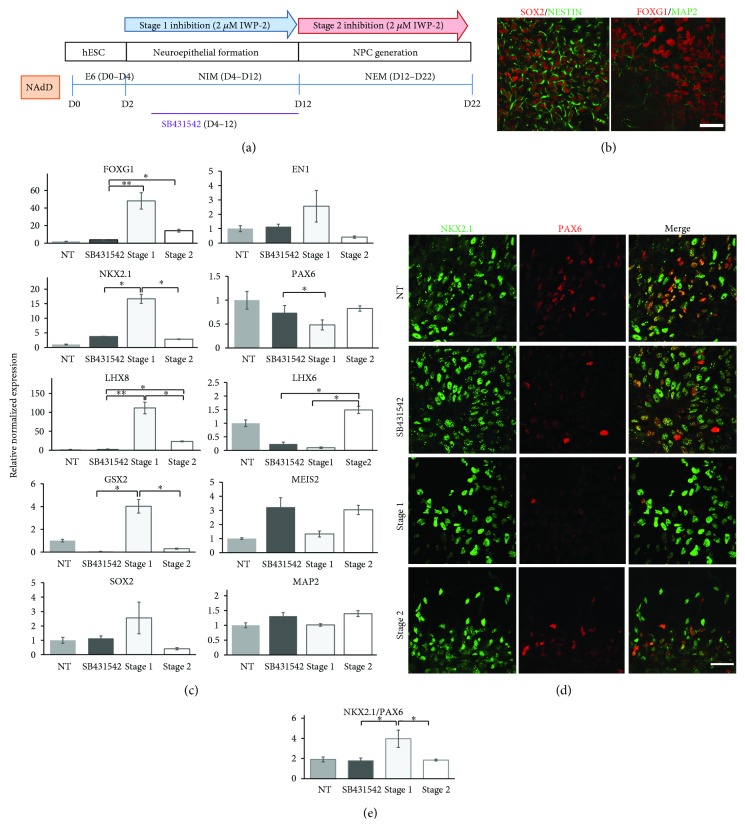
Effect of timing of Wnt inhibition on generation of MGE progenitors in the NadD protocol. (a) Schematic timeline of the NAdD protocol showing stages of MGE generation, involved techniques, medium composition, and time of treatment with SB431542 and IWP-2 and medium change. (b) Confocal images of NPCs double stained with SOX2/NESTIN and FOXG1/MAP-2 antibodies. NPC collected at D22 and plated on 8-well chambers were fixed and stained two days later. Scale bar: 100 *μ*M. (c) Quantitative RT-PCR analysis of various markers (expression relative to nontreated cells, NT = 1) in NPC on D22 of directed differentiation. Data are presented as mean ± SEM. ^∗^ *p* < 0.05 and ^∗∗^ *p* < 0.001 difference between treatment with SB431542 alone and SB431542 supplemented with IWP-2 and between Wnt inhibition in stage 1 and stage 2. (d) Immunocytochemistry analysis of NPC for ventral, NKX2.1, and dorsal, PAX6, forebrain markers in response to treatment with SB431542 alone and SB431542 supplemented with Wnt inhibition during the NSC stage (stage 1) and/or NPC stage (stage 2). Scale bar: 100 *μ*M. (e) Quantification of data in (d). Graph showing the NKX2.1/PAX6 ratio in untreated cells (NT) and cells treated with SB431542 alone and SB431542 supplemented with Wnt inhibition during the NSC stage (stage 1) and/or NPC stage (stage 2). Data are presented as mean ± SD. ^∗^ *p* < 0.05 difference between treatment with SB alone and with SB + IWP-2.

The EB-based nonadherent protocol, NAdD (modified from [21]), with Wnt inhibition during NSC and NPC differentiation was studied.  Figure 5(a) represents a schematic timeline of MGE progenitor creation, medium change, SMAD inhibition (SB 431542), and timing of Wnt inhibition. Throughout the generation of NPC, the cells were detached, making the protocol technically challenging. Of note, in this protocol SHH is not added exogenously. It was reasoned that the 3D environment creates the necessary gradient of SHH [21]. Inhibition of the SMAD pathway by SB 431542 is necessary and sufficient for successful neuronal induction and ventralization of neuroepithelia [21]. In our experiments, the level of secreted SHH during neural induction/rostralization was quantified (Supplementary data, Figure S3A). Gene expression analysis confirmed that at the NSC stage, the level of *SHH* is the highest in cells undergoing neural differentiation through the NAdD protocol compared to the RD and AdD strategies (Supplementary data, Figure S3B). The NAdD approach results in a population of forebrain progenitors as confirmed by double immunostaining with SOX/NESTIN and FOXG1/MAP2 (Figure 5(b)).

Gene expression analysis revealed differences in progenitors obtained by the NAdD protocol compared to the RD and the AdD protocols (Supplementary data, Table S3). Inhibition of Wnt signaling in stage 1 and stage 2 had a significant effect on differentiation along the R-C neuraxis. The expression level of forebrain marker *FOXG1* was increased around 12 times by IWP-2 treatment during neuronal initiation/rostralization (stage 1) and by about 3.6 times when inhibition of Wnt signaling occurred during stage 2 (Figure 5(c), top row; Supplementary data, Table S3) compared to SMAD inhibition by SB 431542. *EN1* expression was low in untreated samples, and Wnt inhibition did not affect it (Figure 5(c), top row; Supplementary data, Table S3). Similar to the RD protocol, the expression of *HOXC6*, a hindbrain marker, was low in untreated cells and treatment with IWP-2 further diminished it (Supplementary data, Figure S1).

Inhibition of Wnt signaling significantly changed the expression levels of ventral markers *NKX2.1*, *LHX8*, and *LHX6*. Expression of dorsal marker *PAX6* decreased in the cells treated with SB431542 compared to untreated controls; stage 1, but not stage 2, Wnt inhibition further decreased the level of *PAX6* expression in NPC (Figure 5(c), second row; Supplementary data, Table S3). Importantly, stage 1 inhibition showed a more prominent effect on *NKX2.1* and *LHX8* expression levels than did stage 2. Expression of *LHX8* has increased more than 35 times in the cells treated with IWP-2 in stage 1 and about 7 times if the inhibitor was added to the cells during stage 2 of neural differentiation (Figure 5(c), third row; Supplementary data, Table S3). While expression of *NKX2.1* has been increased 4.5 times by Wnt inhibition at stage 1, it remained unchanged if the inhibitor was applied during stage 2 (Figure 5(c), second row; Supplementary data, Table S3). In contrast, the expression level of *LHX6* was downregulated in stage 1 inhibition, but was significantly upregulated in stage 2 inhibition compared to treatment with SB431542 alone (Figure 5(c), third row; Supplementary data, Table S3). In addition to the significant increase in gene expression of MGE markers, early treatment of cells with IWP-2 (stage 1) also led to a significant increase in LGE marker *GSX2*, but not *MEIS2* (Figure 5(c), fourth row; Supplementary data, Table S3). Similar to the RD and AdD approaches, expression levels of *SOX2* and *MAP2* remained stable during cell treatment (Figure 5(c), bottom row; Supplementary data, Table S3).

Immunocytochemistry analysis of NPC and counts of NKX2.1^+^ and PAX6^+^ cells confirmed that the NAdD protocol enabled generation of ventral forebrain progenitors (Figure 5(d); Supplementary data, Figure S2). The NKX2.1/PAX6 ratio of untreated cells and of cells treated with SB 431542 was 2, showing that two-thirds of NPC are MGE progenitors. Treatment with the IWP-2 inhibitor at the stage of neural induction (stage 1) caused a further increase in this ratio to 4 (Figure 5(e)). Stage 2 inhibition did not affect the NKX2.1/PAX6 ratio (Figure 4(e)).

### 3.4. Comparison of Experimental Models

Purity of MGE progenitors (expressed as a percentage of NKX2.1^+^ cells in the total number of generated NPC (NKX2.1^+^ cells plus PAX6^+^ cells)) was contrasted between the three distinct differentiation strategies and between stage 1 and stage 2 inhibition within each protocol (Table 1). When compared to baseline conditions (treatment with SHH alone in the RD and AdD protocols and treatment with SB431542 in the NAdD protocol), stage 1 inhibition significantly increased the purity of MGE progenitors from 56% to 78% in the RD (*p* = 0.022), from 64% to 79% in the NAdD (*p* = 0.035), and from 55% to 71% in the AdD differentiation systems. At the same time, Wnt inhibition at stage 2 resulted in a 20% increase in purity of the MGE progenitor culture in both the RD and AdD protocols (from 56% to 76% (*p* = 0.027) and from 55% to 75% (*p* = 0.045), resp.) in comparison with the baseline. Purity of MGE progenitors was not affected by stage 2 inhibition in the NAdD differentiation system.

It is important to note that while stage 1 inhibition significantly increased the purity of MGE progenitors compared to the baseline conditions in all three differentiation systems, treatment with the Wnt inhibitor at the stage of neuronal induction/rostralization substantially lowered the total number of MGE progenitors, especially in the EB-based protocols.

## 4. Discussion

Differentiation of human pluripotent stem cells into MGE progenitors represents the first step in the *in vitro* development of GABA interneurons and BFCN, two pertinent cell types in numerous neuropsychiatric diseases. Optimization of this processs is needed in order to develop reproducible cell-based models.

Several differentiation strategies have been succesfully employed to create MGE progenitors from human pluripotent stem cells including the RD [15, 16], AdD [12, 20], and NAdD [21] protocols. As MGE progenitors arise from the most ventral part of the forebrain, inhibition of Wnt signaling is often incorporated into the strategy. However, the timing of Wnt inhibition substantially varies between the published protocols. Inconsistency in timing and a lack of standardized outcomes regarding purity make comparison challenging. This information is crucial for selecting strategies for downstream applications and for optimizing the signal-to-noise ratio. We employ three different experimental strategies including Wnt inhibition at two distinct timepoints: in the NSC or NPC stages.

The initial steps of the EB-based differentiation protocols, both the RD and the NAdD, follow the neural induction principle *in vivo.* Detachment of hESC/iPSC from the matrix and the removal of serum and self-renewal components from culturing media lead to a differentiation toward three germ layers and result in EB formation. Importantly, these 3D conditions support neural and restrain mesodermal and endodermal differentiation [27]. During culturing, intrinsic production of BMP and FGF inhibitors eliminates the necessity of exogenous growth factors [28]. The need for extrinsic Wnt inhibition during the NSC stage of MGE generation depends on technical details and medium composition of a chosen EB-based protocol, and thus, the reports are conflicting. For example, Liu et al. demonstrate that Wnt inhibition is not required for *in vitro* generation of 90% pure MGE-like progenitor culture in the RD protocol [16]. In contrast, various Wnt inhibitors are added to neural induction media if rosette formation/picking is not a part of the EB-based protocol [13, 18, 19]. Meanwhile, inhibition of Wnt signaling with DKK1 during the NPC stage enhances MGE formation [15].

In the RD approach, both stage 1 and stage 2 inhibitions promote expression of MGE markers. Based on NKX2.1^+^ cell count, the purity of MGE progenitors increases from 56% in the baseline (treatment with SHH alone) to 78% in stage 1 and to 76% in stage 2 of Wnt inhibition. However, early treatment with the Wnt inhibitor (stage 1) results in a lower number of generated MGE progenitors. Increase in purity of MGE progenitors caused by inhibition of Wnt signaling during NPC stage is consistent with Li et al. [15].

Similar to the RD protocol, inhibition of the Wnt pathway during the NSC stage is associated with a lower number of cells due to early block of proliferation in comparison with treatment with a SMAD inhibitor, SB421543, alone in the NAdD protocol. On the other hand, it improves purity of MGE progenitors from 65% at baseline to 79% in stage 1. Stage 2 inhibition has no statistically significant impact on purity of MGE progenitors due to high variability in sizes between individual neurospheres. It is worthwhile to note that this is the first attempt to supplement the NAdD protocol previously used for BFCN generation [21] with Wnt inhibition.

While EB-based differentiation protocols better mirror neural differentiation *in vivo*, EB formation followed by manual rosette picking is labor-intensive and time-consuming and has limited scalability. Furthermore, EBs are very sensitive to changes from experiment to experiment, which in turn leads to variability in the quality of neuroepithelia. To improve yield and consistency of NSC and at the same time reduce technical challenges, the adherent “dual SMAD inhibition protocol” was introduced in 2009 [22]. Since then, several other adherent protocols have been developed [29, 30]. In the current study, to generate NSC from hESCs, we use a rapid derivation adherent protocol (AdD) based on the use of a commercially available neural induction medium, which reduces the variability between the experiments [23]. Similar to the RD protocol, SHH is used to ventralize neuroepithelia. The protocol is complemented by Wnt inhibition at NSC (stage 1) or NPC (stage 2) differentiation stage in our experiments. While inhibition of the Wnt pathway during the NSC stage has already been succesfully employed to generate MGE progenitors [12, 20], this is the first report of inhibiting the Wnt pathway at the stage of NPC (MGE) generation by using the AdD protocol. Whereas both stages improve the purity of MGE progenitors, stage 2 inhibition appears superior to stage 1 and results in a higher number of MGE progenitors. Our results of stage 1 inhibition are in agreement with those previously reported [12, 20]. However, due to multiple variables in each protocol, including use of SMAD inhibitors, FGF8, or different induction media, direct comparison is limited.

MGE progenitors give rise to two neuronal subtypes—GABA interneurons and BFCN. The ratio of GABA interneurons to BFCN is significatly shifted in favor of GABA interneurons; 86%—GABA versus 14%—BFCN [1]. Several strategies have been applied to generate a higher amount of BFCN from MGE progenitors, including coculturing MGE progenitors with astrocytes [16], transfecting MGE progenitors with *LHX8* and *GBX1* transcription factors or treating them with BMP9 [31], or with high a concentration of nerve growth factor, NGF [32]. Here, we show that in both EB-based protocols (the RD and the NAdD), the timing of Wnt inhibition results in opposite effects of *LHX8*/*LHX6* expression. While inhibition in the NSC stage (stage 1) upregulates *LHX8*, the transcription factor needed for BFCN differentiation [25], inhibition in the NPC stage (stage 2) increases the expression level of *LHX6*, the transcription factor required for GABA interneurons [26], suggesting that timing of Wnt inhibition is a viable strategy to promote BFCN versus GABA interneuron differentiation.

In summary, stage 1 inhibition substantially increases the purity of MGE progenitors. Purity of the cell-based model systems determine the signal-to-noise ratio in downstream application, thus improving homogeneity in an active research field. The profound contribution of the MGE-derived cell types to human neuropsychiatric diseases and the current need of robust models make these efforts a priority. Further systematic protocol development strategies are needed to optimize the models.

## Figures and Tables

**Table 1:** Comparison of purity of MGE progenitors obtained in the three differentiation approaches.

Protocol	RD	AdD	NAdD
	Purity (%)	Purity (%)	Purity (%)
Baseline^∗^	56 ± 1.89	56 ± 1.81	64 ± 1.53
Stage 1	78 ± 3.42	71 ± 2.54	79 ± 2.16
Stage 2	76 ± 2.29	75 ± 2.00	65 ± 0.50

^∗^Baseline in the RD and AdD protocols represents treatment with SHH alone. Baseline in the NAdD protocol represents treatment with SB431542.

## Supplementary Materials

Supplementary Materials Table S1: primer sequences used for RT-qPCR. Table S2: anti-human antibodies used for immunocytochemistry. Table S3: relative gene expression in MGE generated in the RD, AdD, and NAdD strategies. Figure S1: quantitative RT-PCR analysis of a hindbrain marker HOXC6. Figure S2: Wnt inhibition changes the expression of dorsal (PAX6) and ventral (NKX2.1) forebrain progenitors in the three differentiation protocols. Figure S3: the level of SHH during neuronal induction/neuroepithelium generation.
