# Supplementary material for: Timing of Wnt Inhibition Modulates Directed Differentiation of Medial Ganglionic Eminence Progenitors from Human Pluripotent Stem Cells
Source: Stem Cells Int. 2018 Jun 27;2018:3983090. doi: 10.1155/2018/3983090 (PMC6040240; doi:10.1155/2018/3983090)
Supplement: Supplementary Materials — Table S1: primer sequences used for RT-qPCR. Table S2: anti-human antibodies used for immunocytochemistry. Table S3: relative gene expression in MGE generated in the RD, AdD, and NAdD strategies. Figure S1: quantitative RT-PCR analysis of a hindbrain marker HOXC6. Figure S2: Wnt inhibition changes the expression of dorsal (PAX6) and ventral (NKX2.1) forebrain progenitors in the three differentiation protocols. Figure S3: the level of SHH during neuronal induction/neuroepithelium generation. [file 3983090.f1.pdf]

Table S1. Primer Sequences used for RT-qPCR

Gene	Forward Sequence	Reverse Sequence	Reference
FOXG1	AGAAGAACGGCAAGTACGAGA	TGTTGAGGGACAGATTGTGGC	Bissonnette et al., 2011
EN1	CCCTGGTTTCTCTGGGACTT	GCAGTCTGTGGGGTCGTATT	Yan et al., 2005
HOXC6	CACCGCCTATGATCCAGTGAGGCA	GCTGGAACTGAACACGACATTCTC	Moon et al., 2012
PAX6	ACCACACCGGTTTCCTCCTTCACA	TTGCCATGGTGAAGCTGGGCAT	Imaizumi et al., 2015
PAX6	ACAGATCTACGCCAGCGACT	CATGGTGTCGTAGTGGATGC	Liu et al., 2013
NKX2.1	CGCATCCAATCTCAAGGAAT	CAGAGTGTGCCCAGAGTGAA	Liu et al., 2013
LHX8	CCAAAACCAGCAAAAAGAGC	TGGCGTGCTCTACAATTCTG	Liu et al., 2013
LHX6	ACAGATCTACGCCAGCGACT	CATGGTGTCGTAGTGGATGC	Liu et al., 2013
GSX2	AGGAGAGAAGGGGACTCAGC	CGGACACTGACATCACCAAC	Vergano-Vera et al., 2009
MEIS2	GACCACGATGATGCAACC	CCTGTGTCTTGCGCTAACTG	Zha Y et al., 2014
SOX2	ACACCAATCCCATCCACACT	GCAAACTTCCTGCAAAGCTC	Sundberg et al., 2011
MAP2	AATAGACCTAAGCCATGTGACATCC	AGAACCAACTTTAGCTTGGGCC	Muratore et al., 2014
SHH	GAG TCT CTG CAC TAC GAG GG	CGA GTT CTC TGC TTT CAC CG	

Table S2. Anti-human antibodies used for immunocytochemistry

Antibody	Species	Supplier	Dilution
α - SOX2	Rabbit	Cell Signaling Technology	1:400
α - NESTIN	Mouse	Millipore	1:1000
α - FOXG1	Rabbit	Abcam	1:1000
α - MAP2	Mouse	R&D Systems	1:250
α - NKX2.1	Rabbit	Abcam	1:250
α - PAX6	Sheep	R&D Systems	1:200

Table S3. Comparison of the relative gene expression (ΔCT) in MGE generated in the RD, AdD, and the NAdD strategies

Differentiation protocol	RD		AdD		NAdD		
Treatment	Relative quantity	SE	Relative quantity	SE	Relative quantity	SE	Gene
NT	0.898	0.036	0.674	0.026	0.661	0.036	GAPDH
Baseline *	0.757	0.097	0.816	0.056	0.814	0.248	
Stage 1	0.498	0.049	0.502	0.044	0.461	0.133	
Stage 2	0.642	0.043	0.888	0.047	0.798	0.053	
NT	0.960	0.132	0.161	0.070	0.040	0.003	FOXG1
Baseline	0.561	0.193	0.276	0.085	0.050	0.005	
Stage 1	0.496	0.038	0.361	0.013	0.925	0.087	
Stage 2	0.579	0.018	0.409	0.120	0.482	0.093	
NT	0.108	0.002	0.115	0.016	0.114	0.002	EN1
Baseline	0.029	0.004	0.249	0.043	0.139	0.009	
Stage 1	0.010	0.001	0.373	0.061	0.230	0.010	
Stage 2	0.029	0.022	0.063	0.008	0.054	0.006	
NT	0.016	0.004	0.021	0.002	0.081	0.003	NKX2.1
Baseline	0.200	0.012	0.232	0.051	0.322	0.005	
Stage 1	0.960	0.084	0.202	0.013	0.667	0.047	
Stage 2	0.304	0.033	0.340	0.023	0.306	0.023	
NT	0.009	0.004	0.037	0.004	0.017	0.013	LHX8
Baseline	0.046	0.012	0.236	0.044	0.051	0.004	
Stage 1	0.913	0.084	0.044	0.013	0.915	0.092	
Stage 2	0.228	0.033	0.333	0.023	0.374	0.013	
NT	0.033	0.016	0.031	0.004	0.165	0.023	LHX6
Baseline	0.276	0.008	0.249	0.044	0.051	0.004	
Stage 1	0.014	0.006	0.214	0.033	0.038	0.009	
Stage 2	0.278	0.025	0.681	0.073	0.244	0.023	
NT	0.403	0.009	0.308	0.048	0.382	0.063	PAX6
Baseline	0.311	0.043	0.256	0.003	0.266	0.040	
Stage 1	0.068	0.018	0.217	0.013	0.138	0.010	
Stage 2	0.085	0.030	0.139	0.013	0.209	0.034	
NT	0.092	0.018	0.257	0.066	0.496	0.043	GSX2
Baseline	0.518	0.061	0.486	0.034	0.026	0.005	
Stage 1	0.013	0.014	0.093	0.011	0.256	0.015	
Stage 2	0.202	0.025	0.183	0.009	0.163	0.024	
NT	0.113	0.002	0.104	0.023	0.029	0.002	MEIS2
Baseline	0.016	0.001	0.162	0.005	0.109	0.003	
Stage 1	0.007	0.001	0.278	0.025	0.021	0.001	
Stage 2	0.092	0.018	0.126	0.019	0.086	0.003	
NT	0.903	0.094	0.685	0.101	0.504	0.015	MAP2
Baseline	0.794	0.058	0.765	0.051	0.664	0.056	
Stage 1	0.558	0.120	0.604	0.033	0.314	0.026	
Stage 2	0.574	0.022	0.808	0.040	0.502	0.027	
NT	0.683	0.016	0.685	0.101	0.660	0.139	SOX2
Baseline	0.794	0.080	0.705	0.096	0.891	0.014	
Stage 1	0.498	0.062	0.534	0.006	0.416	0.024	
Stage 2	0.574	0.041	0.728	0.040	0.753	0.116	

* Baseline in the RD and the AdD protocols represents treatment with SHH alone

* Baseline in the NAdD protocol represents treatment with SB431542

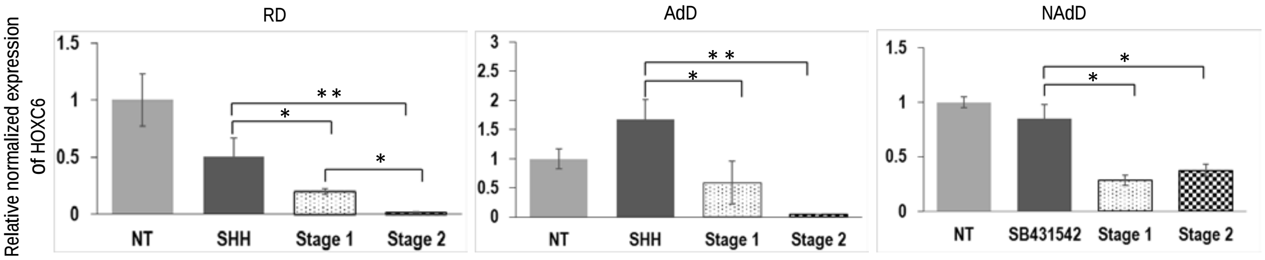

Supplementary figure S1. Quantitative RT-PCR analysis of a hindbrain marker HOXC6 (expression relative to non-treated cells, NT = 1) in NPC generated in the RD, AdD, and NAdD protocols. Data are presented as mean ± SEM. * P < 0.05 and ** P < 0.001 difference between treatment with Shh alone (in the RD and AdD protocols) or SB431542 alone, (in the NAdD protocol)] and Wnt inhibition supplemented during NSC stage (Stage 1) or NPC stage (Stage 2).

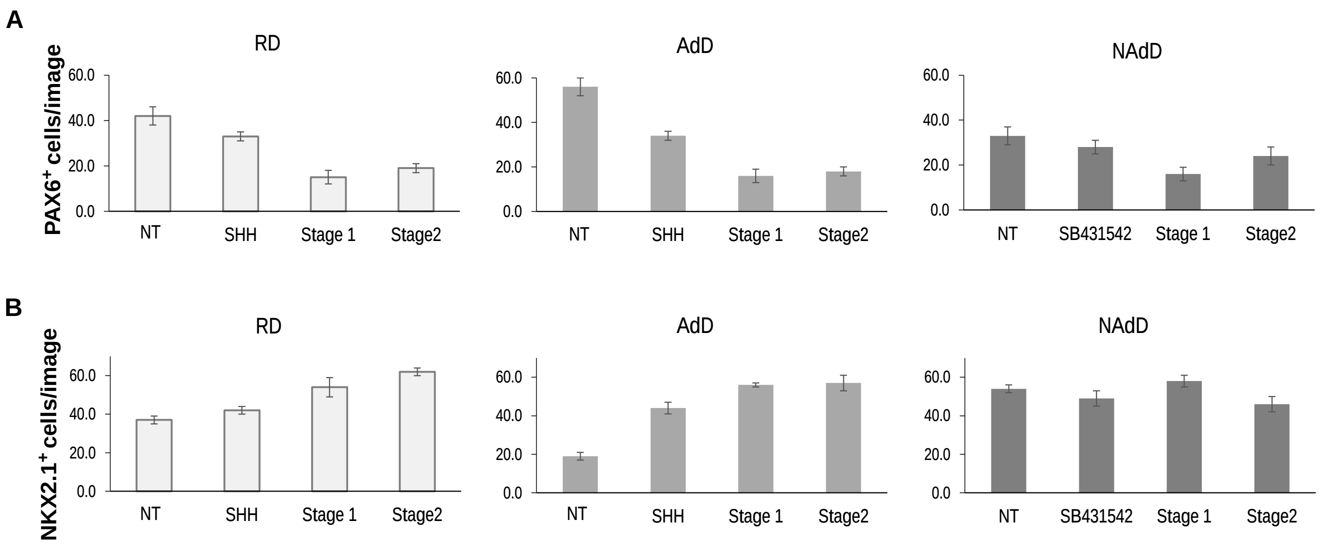

Supplementary Figure S2. Wnt inhibition changes expression of dorsal (PAX6) and ventral (NKX2.1) forebrain progenitors in the three differentiation protocols. Decrease in number of $PAX6^{+}$ cells **(A)** and increase in number of $NKX2.1^{+}$ cells **(B)** in NPC treated with SHH and/or SB431542 alone and in combination with IWP-2 during NSC (Stage 1) and NPC (Stage 2) stages of neural differentiation. Data are presented as mean±SD.

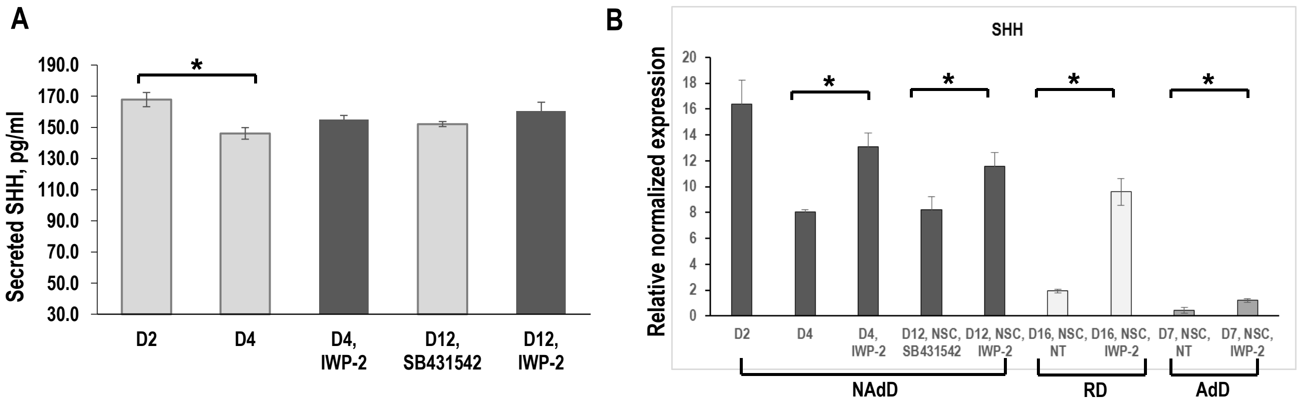

Supplementary Figure S3. The level of SHH during neuronal induction/neuroepithelia generation.
A - The level of SHH in culture media at D2, D4, and D12 secreted by the cells undergoing NAdD differentiation with or without Wnt inhibition. Significant decrease in the level of SHH between D2 and D4 is observed in the samples without IWP-2. Wnt inhibition is needed to sustain SHH level.
B – *SHH* gene expression during neuroepithelia generation and at the stage of NSC in the three differentiation strategies. The highest level of *SHH* is detected in the NAdD protocol. The expression of *SHH* is higher in all protocols in the presence of IWP-2.
